# Sarcopenia Is Associated With Increased Risk of Abnormal Sleep Duration in the Older People: A 10‐Year Cohort Study From China

**DOI:** 10.1002/jcsm.70017

**Published:** 2025-07-26

**Authors:** Yimi Wang, Weixin Chen, Chun Wang, Lingzhi Li, Bingqing Bi, Shugang Li, Hao Wu

**Affiliations:** ^1^ School of Medicine Shihezi University Shihezi China; ^2^ School of Public Health Capital Medical University Beijing China; ^3^ School of General Practice and Continuing Education Capital Medical University Beijing China

**Keywords:** aged, cohort studies, risk factors, sarcopenia, sleep duration

## Abstract

**Background:**

With the acceleration of the aging process, health issues of older people have gradually become one of the most important research topics. The older people experience significant reductions in muscle mass and physical performance. Cross‐sectional studies have found an association between the sarcopenia and sleep duration. Due to lack of evidence of longitude study, it is necessary to investigate the association between sarcopenia and sleep duration in the older people.

**Methods:**

This study utilized longitudinal data including 2061 older people from China Health and Retirement Longitudinal Study 2011–2020. Diagnosis was made based on the Asian Working Group for Sarcopenia criteria 2019. There were 246 participants with sarcopenia, 628 participants with possible sarcopenia and 1187 participants with nonsarcopenia. Kaplan–Meier curve, log‐rank test and Cox proportional risk model were used to explore the relationship between sarcopenia exposure and clinical outcome of abnormal sleep duration. Three models adjusting for different factors were built, and hazard ratios (HRs) were calculated. Restricted cubic spline analyses explored the exposure–response relationship between sarcopenia components and abnormal sleep duration incidence, while receiver operating characteristic curve was used to assess their predictive power.

**Results:**

The incidences of short or long, decreased sleep duration of the sarcopenia and possible sarcopenia groups in the overall population were significantly higher than those of the nonsarcopenia group (respectively, *p* < 0.001). Sarcopenia (HR = 1.240, 95% CI [confidence interval]: 1.023–1.417) was significantly associated with an increased risk of abnormal sleep duration by Cox model analysis with adjusting factors. Results of analyses stratified by sleep duration showed that sarcopenia (HR = 1.566, 95% CI: 1.192–2.057) and possible sarcopenia (HR = 1.286, 95% CI: 1.054–1.570) were positively associated with long sleep duration. Higher ASM (*χ* ^*2*^ = 94.02, *p* < 0.001) and handgrip strength (*χ* ^*2*^ = 94.55, *p* < 0.001), as well as lower five‐time chair stand test (*χ* ^*2*^ = 81.33, *p* < 0.001) showed an exposure‐response relationship with sleep duration. The proportion of the condition of change in sleep duration from normal to < 6 h or > 8 h in the sarcopenia and possible sarcopenia groups was significantly different from the nonsarcopenia group (*p* < 0.05).

**Conclusions:**

Our results suggest that sarcopenia and possible sarcopenia are associated with abnormal sleep duration and the decrease of ASM and handgrip strength, and the increase of the five‐time chair stand test is positively related to abnormal sleep duration, which implies that delaying the development of sarcopenia could be a new pathway to improve sleep health in older people.

## 1 Introduction

As the population aging is accelerating, the health issues of older people have gradually become one of the most important research topics. According to the United Nations projections, by 2070, the number of people aged ≥ 65 is expected to reach 2.2 billion globally, and the number of people aged ≥ 80 is expected to reach 265 million by 2030. Even in countries where the population is still growing rapidly and where the population is relatively young, the number of people aged ≥ 65 is expected to increase over the next 30 years [1]. Studies have shown that older people tend to have more difficulty in falling asleep and maintaining it [2], with up to 50% of older people having problems with sleep disorders [3]. In turn, sleep duration has a significant impact on health and is a risk factor for ailments such as cardiovascular diseases [4] and dyslipidemia [5]. Aging is a lifelong physiological process that begins in early adulthood, and its manifestations (e.g., loss of muscle mass) may be modulated by health behaviours and environmental factors. As people age, they experience a marked reduction in muscle mass and physical performance (Supporting Information s1), which may be related to the manifestations of sarcopenia or presarcopenia [6]. Whereas the aging process affects sleep quality, it has been found that reduced sleep quality and quantity might be associated with sarcopenia [7]. Sarcopenia is an age‐accelerating condition (Supporting Information s2), with prevalence rates of 9% to 45% depending on diagnostic criteria [8]. More than 5% of people aged 60–70 years and 11%–50% aged ≥80 years suffer from sarcopenia [9]. In the UK, the prevalence of sarcopenia is 10% to 27% [8], whereas in Korea, it is 13.1% [10]. Moreover, sarcopenia is also associated with cardiovascular diseases (Supporting Information s3) and neurological diseases (Supporting Information s4). It is an important public health problem for aging societies [11].

It was reported a strong correlation between short sleep duration and possible sarcopenia (OR [odds ratio, OR] = 1.35, 95% CI [confidence interval, CI]: 1.17–1.55), with a prevalence of 53.72% [12]. Ye et al. found the interaction between sleep disorders and sarcopenia [13]. Among Japanese community‐dwelling older people, individuals in the sarcopenia group exhibited a significantly higher prevalence of difficulty initiating or maintaining sleep, affecting 70.9% of cases (*p* < 0.001) [14]. A cross‐sectional study of SarcoPhAge data showed no significant differences between sarcopenia and nonsarcopenia subjects in most components of subjective sleep quality (Supporting Information s5). In summary, existing studies suggest significant co‐occurrence of sarcopenia and sleep disorders, but the direction of the association and the mechanisms still need to be verified by longitudinal studies. Although there are a large number of studies that have elucidated the effects of sleep on sarcopenia, few studies focused on the effects of the sleep disorders induced by sarcopenia. Some cross‐sectional studies have explored the correlation between sleep disorders and sarcopenia, while the longitudinal studies on the link between sleep disorders as well as the changes of sleep duration and sarcopenia have not been reported.

Given that cross‐sectional studies have shown an interaction between sarcopenia and sleep duration, longitudinal studies have also shown that shorter sleep duration is predictive of sarcopenia. In contrast, evidence from longitudinal studies on the relationship between sarcopenia and sleep disorders is lacking. We therefore propose the following hypothesis: Sarcopenia may positively associate with abnormal sleep duration. Based on the China Health and Retirement Longitudinal Study (CHARLS), this study includes older people with normal sleep duration in 2011 who aims to analyse the relationship between the sarcopenia and the change in sleep duration in them from 2011 to 2020 and explores the risk of abnormal sleep duration affected by sarcopenia, to provide a reference for improving sleep quality in the older people.

## 2 Materials and Methods

### 2.1 Study Population

The participants for this study were derived from the CHARLS, a nationally representative longitudinal survey initiated in 2011. It covered 150 county‐level units, 450 village‐level units and 10 000 households with 17 000 people in 28 provinces, and these samples will be followed up every 2 to 3 years. Covering basic personal information, household structure, health status, physical measurements, health service utilization and health insurance and community basics, CHARLS has so far completed four waves of follow‐up data collection, and detailed information about CHARLS has been published [15]. The project was also approved by the Biomedical Ethics Committee of Peking University (IRB 00001052‐11 015), and the data are available on the CHARLS website (http://CHARLS.pku.edu.cn).

**FIGURE 1:**
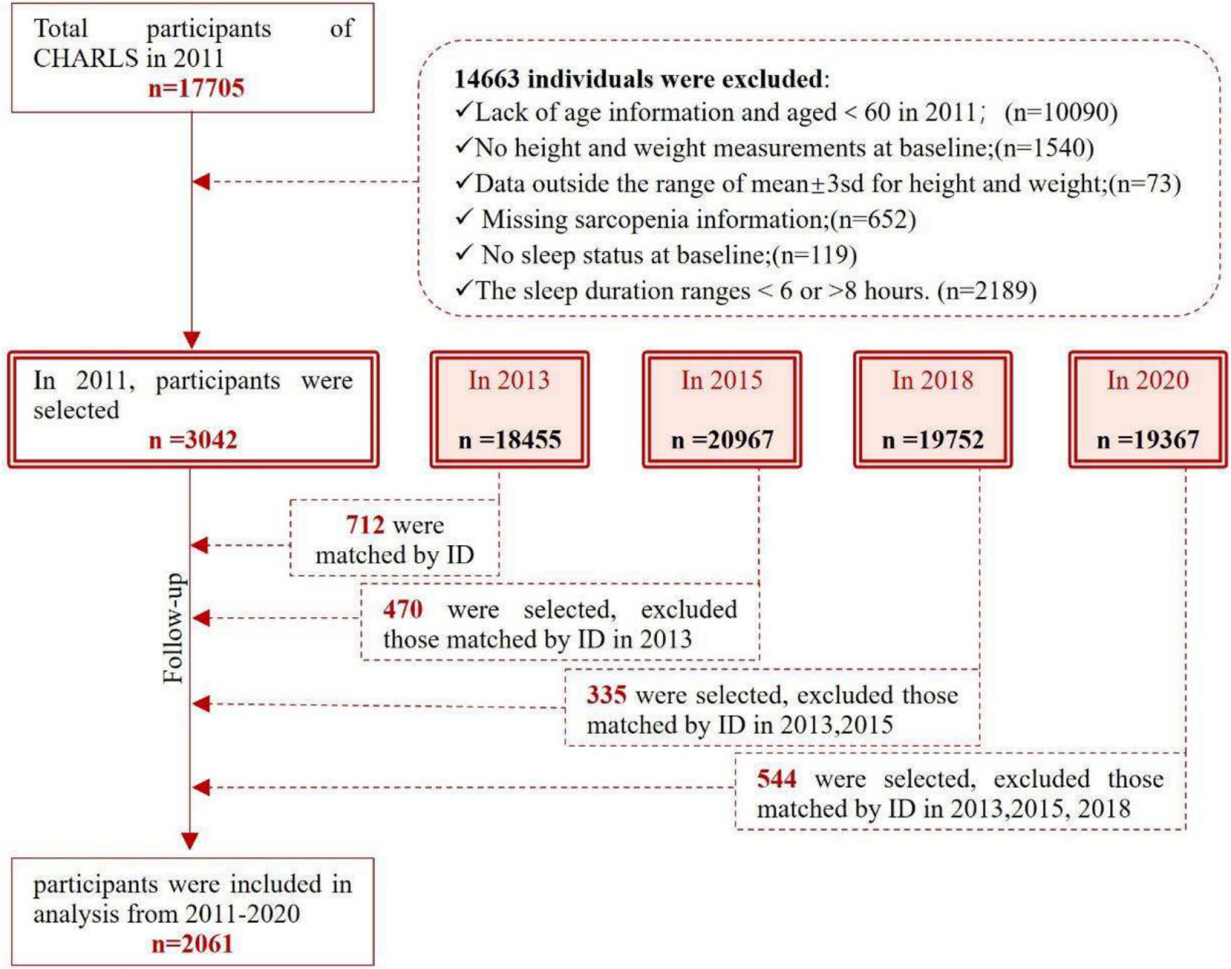
The flow chart of screening for enrolled individuals.

This study used data from the CHARLS from 2011 to 2020 as participants. A total of 17 705 people were surveyed based on the number of people with demographic data at baseline in 2011. We excluded 14 663 people due to (1) lack of age information and age < 60 years, (2) lack of height and weight measurement data, (3) people whose height and weight data were not within the range of ^−^x ± 3 s, (4) lack of diagnostic data for sarcopenia, which includes handgrip strength and the five‐time chair stand test, (5) lack of night sleep duration data and (6) sleep duration in the abnormal range (< 6 h or > 8 h) at baseline; a total of 3042 individuals participated in the baseline analyses. In the longitudinal study, we matched IDs with data from 2013, 2015, 2018 and 2020 to exclude individuals with unmatched IDs and those lacking sleep duration data. Individuals who participated in multiple follow‐ups were selected for inclusion with data from their last follow‐up. Finally, we selected 2061 eligible participants. Detailed information is shown below (Figure 1). The median follow‐up years are 5 years.

### 2.2 Assessment of Sarcopenia

Sarcopenia was the exposure factor in this study. The Asian Working Group for Sarcopenia criteria 2019 (AWGS 2019) algorithm for diagnosing older people was used in this study [16]. In AWGS 2019, skeletal muscle strength was measured by handgrip strength. We used both hands to take the average of the maximum available values. If a participant was unable to take handgrip strength measurements with both hands, the value from the available hand was used. According to AWGS 2019, the threshold for low handgrip strength is < 18 kg for women and < 28 kg for men. Physical performance was tested using the five‐time chair stand test. A time of ≥ 12 s for the five‐time chair stand test was defined as low physical performance. Individuals who could not complete the test were excluded. Estimation of muscle mass using a previously validated formula is as follows [17]:

ASM (Appendicular Skeletal Muscle Mass)

$$=0.193^{*}\text{bodyweight}\;\left(\mathrm{Kg}\right)+0.107^{*}\text{height}\;\left(\mathrm{cm}\right)$$

$$-4.157^{*}\mathrm{sex}\;\left(1\;\text{for male},2\;\text{for female}\right)$$

$$-0.037^{*}\mathrm{age}\;\left(\mathrm{yr}\right)-2.631.$$

After estimating the ASM value, the muscle mass index was calculated using the ASM divided by the square of height in metres (ASMI = ASM/height^2^). Therefore, the cutoff values for the Appendicular Skeletal Muscle Mass Index (ASMI) used in this study were based on the lowest 20% percentile of the study population; low muscle mass was defined if the ASMI was < 6.94 kg/m^2^ for men or < 5.16 kg/m^2^ for women. Sarcopenia was evaluated when muscle mass, muscle strength or physical performance were low, and possible sarcopenia was defined if muscle strength was low or physical performance was low.

### 2.3 Assessment of Sleep Duration

Abnormal sleep duration was the primary outcome of this study [18]. The length of sleep at night was based on participants' subjective recollections, with the questionnaire asking “During the past month, how many hours of actual sleep did you get at night (average hours for one night)? (This may be shorter than the number of hours you spend in bed.)”. And then based on the participants' responses, sleep duration was divided into three categories [19]: short sleep duration (< 6 h), medium sleep duration (6–8 h) and long sleep duration (> 8 h) (Supporting Information s6). The amount of change in sleep duration was the difference between the sleep duration obtained using the last follow‐up visit minus the sleep duration in 2011, with > 0 defined as an increase, < 0 as a decrease and = 0 as no change.

### 2.4 Covariates

Based on the reported evidence, we considered sociodemographic characteristics and health‐related factors in our analyses. Sociodemographic characteristics included age, gender, area of residence (city or rural), current marital status (married or unmarried) and education level, with a cutoff of junior high school graduation dividing participants into two groups. Health‐related factors included current smoking status, current alcohol status and prevalence of chronic diseases. Chronic diseases included the following: hypertension, dyslipidemia, diabetes or high blood sugar, cancer, chronic lung diseases, liver diseases, Heart attack, stroke, kidney diseases, stomach or other digestive diseases, Emotional nervous or psychiatric disorders, memory‐related diseases, arthritis or rheumatism and asthma. The grouping was based on the number of chronic diseases suffered, categorized as nonchronic disease, one chronic disease and two or more chronic diseases.

### 2.5 Statistical Analysis

Categorical variables were described with frequencies and percentages, and the differences between the sarcopenia and possible sarcopenia groups and changes in sleep duration as well as changes in sleep conditions were analysed by chi‐square test. Quantitative variables were described as mean ± standard deviation with normal distribution and described by median and interquartile for the variables with abnormal distribution. Participants' characteristics were compared using one‐way ANOVA, Kruskal–Wallis H test and chi‐square tests. The Kaplan–Meier survival analysis was used to compare the abnormal sleep duration incidence curves and log‐rank tests to compare differences in sleep duration incidence among groups. Cox regression was employed to estimate the longitudinal associations of the sarcopenia and possible sarcopenia groups with the incidence of abnormal sleep duration and with changes in sleep duration using three models adjusting for age, gender, education, current marital status, habitation, current smoking status, current drinking status and the number of chronic diseases suffered, and the analyses were stratified by gender. Restricted cubic spline (RCS) was used to explore the exposure‐response relationship between the components of sarcopenia and the prevalence of abnormal sleep duration. Receiver operating characteristic (ROC) curve was used for assessing the predictive power of components of sarcopenia on the incidence of abnormal sleep duration. SPSS 27.0 and R4.3.2 were used to conduct the statistical analysis. *p* < 0.05 was considered statistically significant.

## 3 Results

### 3.1 Basic Characteristics of the Study Participants

The study included 2061 participants (1115 males and 946 females) from 28 provinces in China at baseline, of whom 246 participants with sarcopenia and 628 with possible sarcopenia from 2011 to 2020. Patients with sarcopenia were older, lived in the city, had less education, were unmarried and had a lower BMI than those with nonsarcopenia. People with possible sarcopenia had a higher prevalence of hypertension, dyslipidemia, heart attack, kidney diseases and arthritis or rheumatism (Table 1).

**TABLE 1:** Sociodemographic characteristics and critical variables of participants.

Characteristics	Nonsarcopenia (*n*, %) *n* = 1187	Possible sarcopenia (*n*, %) *n* = 628	Sarcopenia (*n*, %) *n* = 246	*F*/*χ* ^2^	*P*
Age	64.0 (62.0, 69.0)	66.0 (62.0, 71.0)	70.0 (66.0, 76.0)	143.878^a^	**< 0.001**
Gender				28.375	**< 0.001**
Male	701 (59.1)	292 (46.5)	122 (49.6)		
Female	486 (40.9)	336 (53.5)	124 (50.4)		
Current married				33.354	**< 0.001**
Married	1029 (86.7)	517 (82.3)	177 (72.0)		
Unmarried	158 (13.3)	111 (17.7)	69 (28.0)		
Habitation				24.651	**< 0.001**
City	1036 (87.4)	584 (93.1)	235 (95.5)		
Rural	150 (12.6)	43 (6.9)	11 (4.5)		
Education				62.586	**< 0.001**
Above junior school	351 (29.6)	101 (16.1)	29 (11.8)		
Others	836 (70.4)	527 (83.9)	217 (88.2)		
Smoking	563 (47.4)	262 (41.7)	108 (43.9)	5.617	0.060
Drinking	435 (36.6)	143 (22.8)	60 (24.4)	42.635	**< 0.001**
Height, m	159.1 (153.0, 165.9)	157.5 (152.0, 163.0)	154.2 (146.1160.8)	67.468^a^	**< 0.001**
Weight, Kg	58.7 (52.0, 66.4)	59.5 (54.3, 68.2)	44.4 (41.0, 48.6)	444.284^a^	**< 0.001**
BMI	23.3 (20.9, 25.5)	24.0 (22.0, 26.6)	19.1 (17.7, 20.0)	485.245^a^	**< 0.001**
Hand strength	33.5 (27.0, 40.0)	26.1 (20.0, 33.4)	22.8 (18.0, 28.1)	358.825^a^	**< 0.001**
ASM	17.57 ± 4.04	17.20 ± 3.78	13.60 ± 3.76	104.918^b^	**< 0.001**
Five‐time chair stand test	9.28 (7.89, 10.50)	13.85 (12.36, 16.40)	13.82 (12.02, 16.28)	1051.258^a^	**< 0.001**
Hypertension	342 (28.9)	232 (37.0)	52 (21.4)	23.464	**< 0.001**
Dyslipidemia	123 (10.5)	85 (13.8)	13 (5.5)	12.645	**0.002**
Diabetes	83 (7.1)	55 (8.8)	10 (4.1)	5.883	0.053
Cancer	8 (0.7)	5 (0.8)	3 (1.2)		0.789^c^
Chronic lung diseases	138 (11.7)	77 (12.3)	39 (16.0)	3.484	0.175
Liver diseases	44 (3.7)	29 (4.6)	4 (1.6)	4.401	0.111
Heart attack	175 (14.8)	125 (20.0)	35 (14.3)	8.885	**0.012**
Stroke	18 (1.5)	21 (3.3)	9 (3.7)	8.187	**0.017**
Kidney diseases	46 (3.9)	39 (6.2)	5 (2.0)	8.913	**0.012**
Stomach diseases	216 (18.2)	113 (18.0)	54 (22.0)	2.150	0.341
psychiatric	13 (1.1)	9 (1.4)	4 (1.6)		0.677^c^
Memory related diseases	22 (1.9)	8 (1.3)	5 (2.0)		1.021^c^
Arthritis or rheumatism	349 (29.4)	239 (38.1)	75 (30.5)	14.649	**< 0.001**
Asthma	44 (3.7)	32 (5.1)	19 (7.8)	7.991	**0.018**

*Note:* “a” represents the chi‐square statistic of the Kruskal–Wallis H test. “b” means ANOVA. “c” stands for Fisher's exact test. F means the statistic of ANOVA. *χ* ^*2*^ represents the statistic of the chi‐square test. BMI means body mass index. ASM means Appendicular Skeletal Muscle Mass. Diabetes is expressed in CHARLS as Diabetes or high blood sugar. Stomach diseases are expressed in CHARLS as Stomach or other digestive diseases. Psychiatric is expressed in CHARLS as Emotional nervous or psychiatric. *p* < 0.05 was considered statistically significant and is marked in bold font in the tables.

### 3.2 Relationship Between Sarcopenia and Incidence of Abnormal Sleep Duration

**FIGURE 2:**
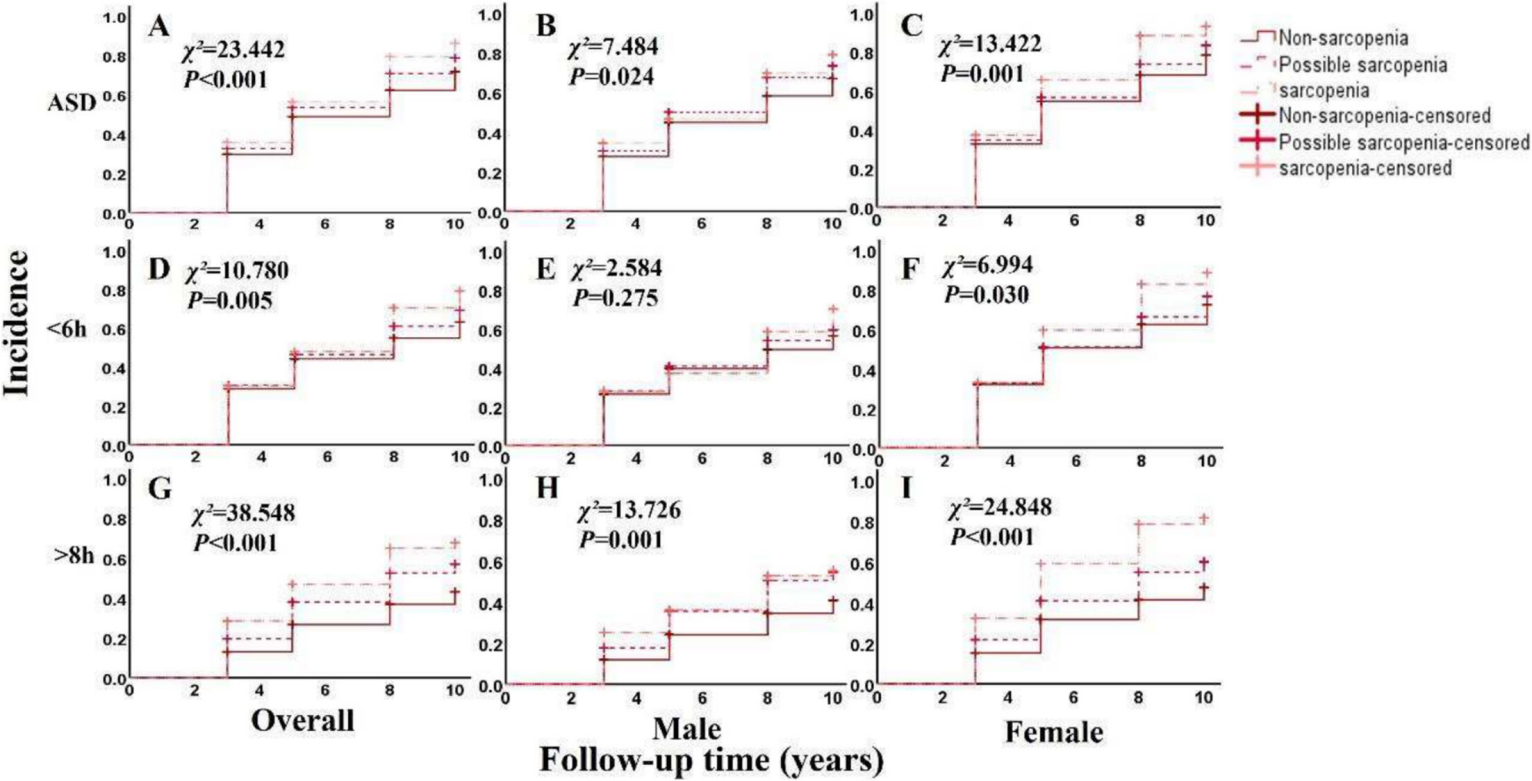
Survival analysis of the incidence of sarcopenia and abnormal sleep duration. *Note:* ASD means abnormal sleep duration (including sleep duration < 6 h and > 8 h). < 6 h stand for sleep duration at the final follow‐up was < 6 h. > 8 h means sleep duration at the final follow‐up was > 8 h. In the case of < 6 h and > 8 h as outcomes, respectively, the sleep duration of 6–8 h was used as a control. Follow‐up time depicts the number of years of follow‐up in 2013, 2015, 2018 and 2020, respectively. *χ* ^*2*^ stands for the statistic of log‐rank test.

**FIGURE 3:**
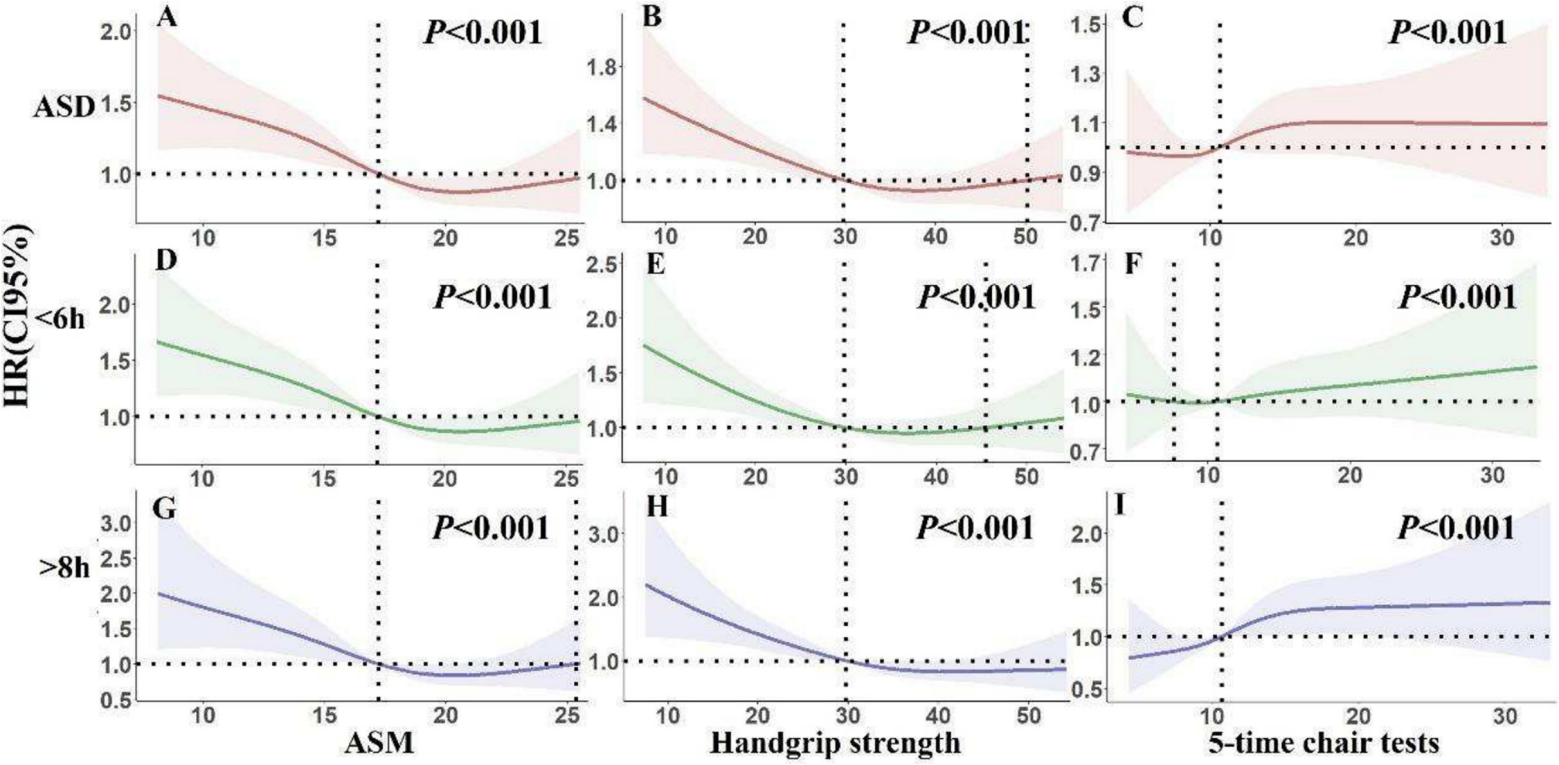
Expose–response curve relationship between components of sarcopenia and incidence of abnormal sleep duration. *Note:* HR means hazard ratio. 95CI% stand for 95% confidence interval. ASD is for abnormal sleep duration. < 6 h stand for sleep duration at the final follow‐up was < 6 h. > 8 h means sleep duration at the final follow‐up was > 8 h. ASM means appendicular skeletal muscle mass. RCS was used to describe expose‐response curve by adjusting factors, including gender, age, education, current marital status and area of residence, smoking, alcohol drinking and number of chronic diseases.

**FIGURE 4:**
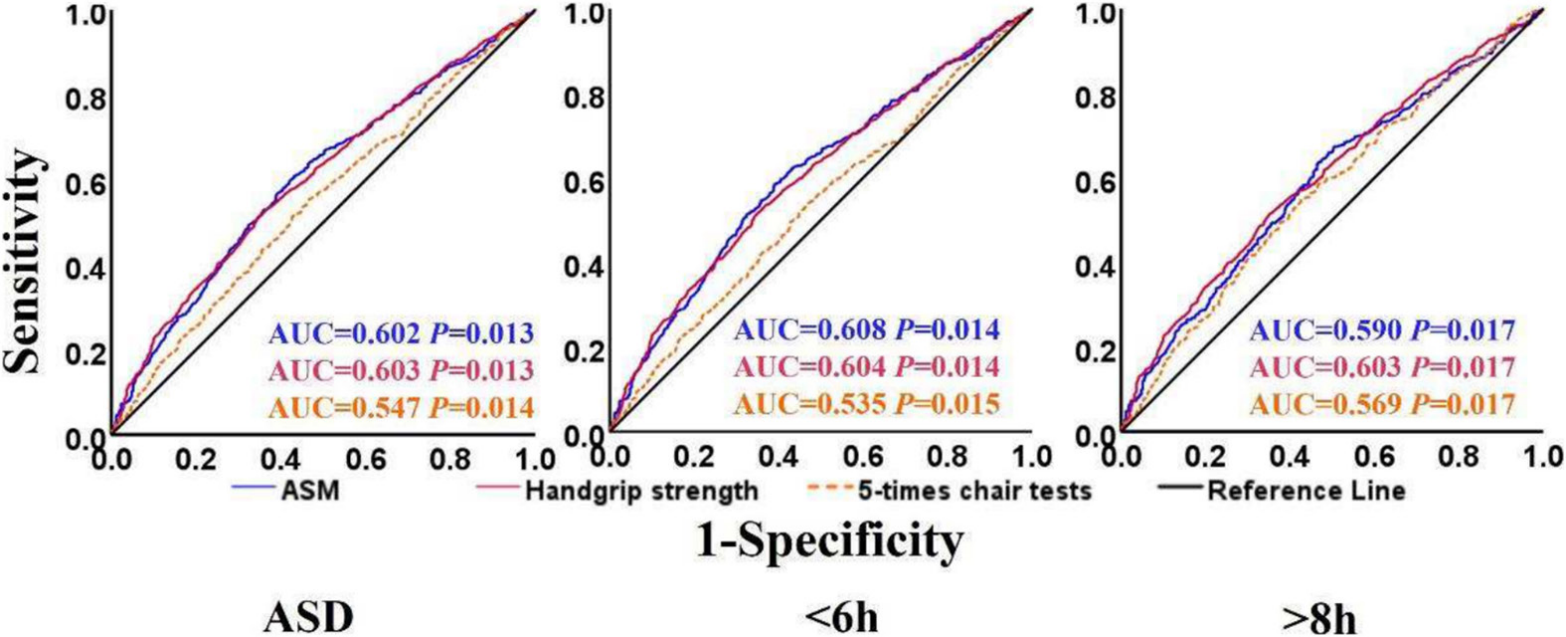
ROC curves of the components of sarcopenia and the incidence of abnormal sleep duration. *Note:* ASD means abnormal sleep duration (including sleep duration < 6 h and > 8 h). < 6 h stands for sleep duration at the final follow‐up was < 6 h. > 8 h depicts sleep duration at the final follow‐up was > 8 h. ASM is for appendicular skeletal muscle mass. AUC indicates area under curve.

Individuals with sarcopenia and possible sarcopenia had a statistically higher incidence of abnormal sleep duration compared with the nonsarcopenia group (both *p* < 0.05, Figure 2) by log‐rank test. In Model 2, the sarcopenia group was positively associated with an abnormal sleep duration (HR [hazard ratio]: 1.269 95% CI: 1.081, 1.491), short (HR: 1.240, 95% CI: 1.013, 1.518) and long (HR: 1.753, 95% CI: 1.341, 2.291). In Model 3, the sarcopenia group was positively associated with abnormal sleep duration (HR: 1.204, 95% CI: 1.023, 1.417) and long (HR: 1.566, 95% CI: 1.192, 2.057), and it is possible that similar results were observed in the sarcopenia group (Table 2). An exposure–response relationship between the components of sarcopenia and the incidence of abnormal sleep duration was observed by the RCS. Higher ASM (*χ* ^*2*^ = 94.02, *p* < 0.001, Figure 3A) and handgrip strength (*χ* ^*2*^ = 94.55, *p* < 0.001, Figure 3B) and lower five‐time chair stand test (*χ* ^*2*^ = 81.33, *p* < 0.001, Figure 3C) were associated with higher incidence of abnormal sleep duration. In contrast, the incidence of handgrip strength with sleep duration < 6 h showed an approximate U‐shaped curve (nonlinear *p* = 0.035 Figure 3E). The AUCs were all greater than 0.5, and it can therefore be assumed that the components of sarcopenia have some predictive value for the incidence of abnormal sleep duration (Figure 4).

**TABLE 2:** The longitudinal association between sarcopenia and different sleep disorders conditions and change.

Outcomes	HR (95% CI)				
	Nonsarcopenia	Possible sarco p enia	*p*	Sarcopenia	*p*
** AS D **					
Mo d e l 1	Refere n ce	1.164 (1.039, 1.3 0 6)	**0 . 009**	1.342 (1. 146, 1 .57 1 )	**< 0 . 001**
Mo d el 2	Ref e r e n ce	1.116 (0.994, 1 .253)	0.063	1.269 (1 .08 1, 1.49 1 )	**0.004**
Mo d el 3	Ref e r e n ce	1 .064 (0 .9 4 6, 1 . 1 97)	0.301	1.204 (1. 023, 1 . 417)	**0.02 6**
**< 6 h**					
Mo d el 1	Refer e n ce	1. 126 (0.977, 1 . 298)	0. 1 02	1.305 (1.070, 1 .59 1 )	**0.008**
Mo d el 2	Referenc e	1 .063 (0.92 1, 1.227)	0.406	1.240 (1. 01 3, 1 .518)	**0.03 7**
Mo d el 3	Refer e n ce	1. 006 (0. 868, 1.1 6 4 )	0. 941	1.1 8 0 (0. 961, 1 .449)	0 .11 4
**> 8 h**					
Mo d e l 1	Refer e n ce	1.467 (1 .208, 1 . 782)	**< 0.001**	1.955 (1 . 506, 2 .537)	**< 0 . 001**
Mo d e l 2	Refe r e n ce	1.387 (1 .139, 1 . 688)	**0.001**	1.753 (1 . 34 1, 2 .291)	**< 0.001**
Mo d el 3	Reference	1. 286 (1.054, 1.570)	**0 . 013**	1.566 (1 . 192, 2 .05 7)	**0 . 001**
** Decr e a s ed **					
Mo d el 1	Reference	1. 067 (0.937, 1.2 1 5)	0.326	1 . 235 (1. 026, 1 .488)	**0 . 02 6**
Mo d el 2	Refere n ce	1. 032 (0.905, 1 . 1 76)	0 . 639	1.191 (0. 985, 1 .439)	0.071
Mo d e l 3	Reference	0.991 (0.867, 1.132)	0.888	1. 134 (0. 9 35, 1 .374)	0 . 20 1
** Incr ea sed **					
Mo d e l 1	Refere n ce	1. 256 (1 .060, 1 .487)	**0 . 008**	1.624 (1 . 294, 2 .039)	**< 0.001**
Mo d e l 2	Refere n ce	1.210 (1 .020, 1.436)	**0 . 029**	1.5 1 6 (1. 202, 1 .9 1 3)	**< 0 . 001**
Mo d el 3	Reference	1.146 (0 .96 4, 1.363)	0. 1 23	1.414 (1. 1 1 5, 1 .793)	**0.004**

*Note:* HR means hazard ratio. 95% CI is for 95% confidence interval. ASD means abnormal sleep duration (including sleep duration < 6 h and > 8 h). < 6 h stands for sleep duration at the final follow‐up was < 6 h. > 8 h means sleep duration at the final follow‐up was > 8 h. Decreased means the difference in sleep duration between baseline and final follow‐up < 0. Increased indicates the difference in sleep duration between baseline and final follow‐up > 0. Model 1 represents that no confounders were included. Model 2 represents adjusted for gender and age. Model 3 represents adjusted for gender, age, education, current marital status and area of residence, smoking, alcohol drinking and number of chronic diseases. The median follow‐up for overall was 5 years. The person‐years of sarcopenia, possible sarcopenia and nonsarcopenia were 1365, 3736 and 7505 years, respectively, and the incidence of sarcopenia, possible sarcopenia, and nonsarcopenia was 72.75%, 63.81% and 55.68%. *p* < 0.05 was considered statistically significant and is marked in bold font in the tables.

### 3.3 Relationship Between Sarcopenia and Changes in Sleep Duration

**FIGURE 5:**
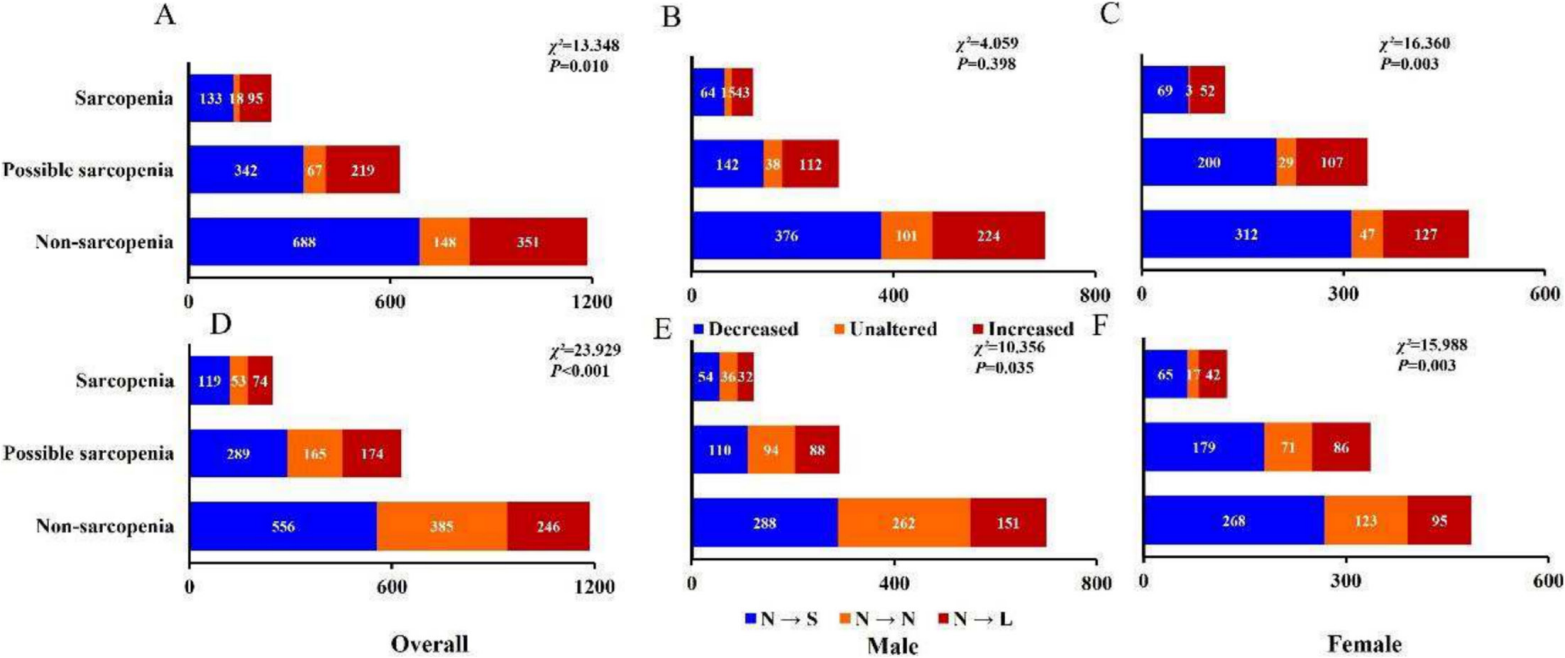
The comparison on proportion of changes in sleep duration and changes in sleep status among different groups (*n*). *Note: χ* ^*2*^ represents the chi‐square test statistic. Decreased means the difference of sleep duration between baseline and final follow‐up < 0. Unaltered stands for the difference of sleep duration between baseline and final follow‐up = 0. Increased indicates the difference of sleep duration between baseline and final follow‐up > 0. “N” stands for normal sleep duration (6–8 h at baseline or final follow‐up). “S” is for short sleep duration (< 6 h at final follow‐up). “L” stands for long sleep duration (> 8 h at the final follow‐up). “→” means normal sleep states in 2011 transferred to sleep states of final follow‐up. Figure 5A–C, respectively, represent the difference on proportion of sleep duration changes (decreased, unaltered and increased) of sarcopenia, possible sarcopenia and nonsarcopenia in overall, male and female population. Figure 5D–F, respectively, represent the difference on proportion of sleep states (N → S, N → N and N → L) of sarcopenia, possible sarcopenia and nonsarcopenia in overall, male and female population.

The incidence of increased sleep duration was higher in both sarcopenia (38.6%) and possible sarcopenia groups (34.9%) than in the nonsarcopenia group (*χ* ^*2*^ = 13.348, *p* = 0.010). After gender stratification, the risk of increased sleep duration in the sarcopenia and possible sarcopenia groups was higher than in the nonsarcopenia group in females (*χ* ^*2*^ = 16.360, *p* = 0.003). However, no statistical difference was observed in males (Figure 5). It was found that the sarcopenia group was positively associated with increase in sleep duration, with or without adjustment for covariates, whereas it was not statistically significant with decreased sleep duration (Table 2).

### 3.4 Relationship Between Sarcopenia and Changes in Sleep Condition

It was found that the incidence of change from normal to < 6 h in the sarcopenia group (48.4%) as well as change from normal to > 8 h in the sarcopenia group (30.1%) and possible sarcopenia group (27.7%) were significantly higher than in the nonsarcopenia group (*χ* ^*2*^ = 23.929, *p* < 0.001). After stratification by gender, the same results were still observed in the male group, whereas the female group had the same results only in the incidence of change from normal to > 8 h group (Figure 5).

## 4 Discussion

In older people, the incidence of abnormal sleep duration is high, which has a great impact on health status, and sarcopenia and possible sarcopenia are associated with sleep duration. Therefore, it is important to study the relationship between sarcopenia and sleep duration in older people. In this study, data from the CHARLS from baseline in 2011 to 2013, 2015, 2018 and 2020 for a total of 10 years of follow‐up surveys were analysed. Longitudinal analyses based on nationally representative data from Chinese older people showed that individuals with sarcopenia were more likely to have sleep duration < 6 h or > 8 h. After adjusting for sex and age, participants with sarcopenia had 1.269 times higher risk of abnormal sleep duration, compared to those with nonsarcopenia. After adjusting for more covariates, it was found that sarcopenia and possible sarcopenia still increased the risk of sleeping > 8 h. Sarcopenia affects sleep duration regardless of its status. In addition, in the effects of muscle mass and muscle strength on the incidence of abnormal sleep duration, respectively, using RCS, we found that ASM, handgrip strength and five‐time chair stand test showed an exposure‐response relationship with the incidence of abnormal sleep duration. These results found a positive association between sarcopenia and abnormal sleep duration.

In this study, the relationship between sarcopenia and abnormal sleep duration is opposite from the previous relationship between sleep disorders and sarcopenia. While previous research on sleep has focused on the central nervous system, there is a growing recognition of the impact of muscles on sleep. Sarcopenia is accompanied by a loss of muscle mass and strength [16]. Poor sleep quality is associated with low muscle mass [20]. Maintaining optimal muscle mass may have beneficial effects on sleep‐related problems [21]. There is a general lack of physical activity in individuals with sarcopenia, and the disorder impairs the ability to perform activities of daily living [22]. In contrast, appropriate resistance training (RT) enhances the redox and inflammatory profile of patients, which may be due to increased NO bioavailability and reduced ADMA concentrations. Low NO bioavailability appears to impair sleep quality in humans and animals as it plays an important role in the regulatory mechanisms of the biological clock and the regulation of sleep brain centres [23]. This may be one of the mechanisms underlying the association of sarcopenia with abnormal sleep duration.

Insulin‐like growth factor 1 (IGF‐1) serves as a key anabolic hormone for maintaining muscle mass [24]. It was reported that female subjects with sarcopenia had significantly lower serum IGF‐1 levels [25]. Existing studies have found that serum IGF‐1 levels are significantly lower in patients with sleep disorders, and IGF‐1 concentration is negatively correlated with apnoea‐hypopnoea index and oxygen saturation index scores and positively correlated with minimum oxygen saturation [26]. Our results showed that sarcopenia and possible sarcopenia were able to increase or decrease sleep duration and change sleep conditions from normal to abnormal conditions in the female population, but this phenomenon was not observed in men. These findings suggest that IGF‐1 may be a potential mechanism for increasing or decreasing sleep duration in sarcopenia, which explains the relationship between changes in sleep status that was only statistically significant in women.

Respiratory sarcopenia is a condition of muscle fibre atrophy and weakness of the respiratory muscles and skeletal muscles throughout the body that occurs with age [27]. Respiratory muscles consist of expiratory and inspiratory muscles. In addition to individual characteristics such as abdominal and thoracic breathing, the respiratory muscles are responsible for a variety of respiratory patterns such as upper chest breathing, jaw breathing and other abnormal breathing patterns, as well as the ventilatory movements that sustain athletic performance and life [28]. Sleep apnea occurs when normal breathing during sleep is interrupted. Respiratory muscle weakness can lead to decreased total sleep time and efficiency [29]. These individuals often exhibit significant sleep fragmentation with frequent nocturnal awakenings (Supporting Information s7) and reduced sleep and rapid eye movement sleep [30]. Thus, the effect of sarcopenia on abnormal sleep duration may also stem from the disease, which needs further studies to verify.

Chronic diseases and health behaviours lifestyle may be other important influencing factors. Previous studies have shown that aging leads to significant physiological and functional changes in circadian rhythms as well as muscle structure and function [31]. In turn, aging is accompanied by a variety of chronic diseases. Skeletal muscle plays a crucial role as a major target of insulin action, and a decline in muscle mass and strength may potentially worsen insulin resistance [32]. Furthermore, Jiang et al. demonstrated a significant association between sarcopenia and multiple metabolic risk factors, including BMI, hypertension, dyslipidemia (elevated triglycerides, reduced HDL cholesterol and increased total cholesterol), and insulin resistance [33]. Alcohol status is significantly associated with all sleep characteristics, and even low levels of alcohol status may affect sleep health, particularly by increasing the risk of snoring and nocturnal chronotypes [34]. In turn, diet can also impact sleep condition, and some nutrients in the diet or their metabolites have been shown to affect sleep health [35]. In our study, it was observed that the sarcopenia group and possible sarcopenia group had a higher prevalence of diseases or conditions such as hypertension, dyslipidemia and alcohol consumption than the nonsarcopenia group. Substantial associations exist between nocturnal features of blood pressure, hypertension and sleep problems [36]. This implies that the worsening of older people's chronic conditions and poor lifestyle habits may exacerbate the effects of sarcopenia on sleep.

Based on current evidence, a multimodal integrated intervention strategy should be implemented to address sarcopenia and its associated sleep problems. Firstly, regarding nutritional optimization, ensuring adequate daily protein intake combined with vitamin D and omega‐3 fatty acid supplementation is recommended, as studies have shown that these interventions significantly enhance muscle synthesis [37]. Secondly, exercise interventions should prioritize RT, supplemented with balance exercises and low‐intensity aerobic activities. Meta‐analyses confirm that this regimen increases muscle mass by 8%–12% and improves sleep efficiency by 15%–20% [38]. For patients with severe conditions, short‐term pharmacological therapy may provide synergistic benefits, though strict monitoring for adverse effects is required [39]. In clinical practice, a multidisciplinary collaborative model integrating geriatrics, rehabilitation and sleep medicine teams is recommended. Regular biomarker monitoring should guide dynamic adjustments to intervention intensity, enabling precision management of the “muscle‐sleep comorbidity.”

This study innovatively modeled the association between sarcopenia and sleep duration. Through a longitudinal cohort design with a 10‐year follow‐up (*n* = 2061, 2011–2020), we systematically revealed a quantitative analysis of the dynamic changes in sleep parameters (including duration fluctuations and state transitions) in an older population. This long‐period multidimensional assessment model breaks through the limitations of previous cross‐sectional studies that only capture static associations. In addition, this study provides a new way of thinking about sarcopenia as a dependent variable inducing a high risk of sleep problems in older people. These important findings are a revolutionary step towards the development of future interventions targeting the effects of sarcopenia on sleep conditions. However, this study has some limitations. Primarily, there may still be other unmeasured confounders that were not adequately considered in this study, such as physical activity, dietary intake and possibly medications. Secondly, the definition of abnormal sleep duration depends on patients' self‐reported questionnaire for measurement, and there is currently some variability in the cutoff values used to define abnormal sleep duration across studies. The definition of abnormal sleep duration that we used may require additional clinical evidence. In addition, CHARLS was designed to conduct follow‐up every 2–3 years, and we were unable to obtain the exact time at which participants experienced an abnormal sleep duration event; the use of survival analyses may not accurately reflect the differences among groups. Thirdly, we used the validated ASMI calculation equation for estimation in the diagnosis of sarcopenia rather than a body composition metre, which may have some bias in the diagnosis of sarcopenia.

## 5 Conclusion

In conclusion, this study suggests that sarcopenia and possible sarcopenia are positively associated with abnormal sleep duration in the older population. Furthermore, there is an exposure–response relationship between components of sarcopenia and the risk of developing abnormal sleep duration. Preventing and delaying sarcopenia help improve sleep health in older people.

## Ethics Statement

CHARLS rounds were approved by the Biomedical Ethics Committee of Peking University. The fieldwork programmer for the current round of the household questionnaire was approved under approval number: IRB00001052‐11015. All participants independently signed informed consent, which was prepared as a PDF file for retention.

## Conflicts of Interest

The authors declare no conflicts of interest.

## Supporting information


**Data S1.** Supplementary Information
