# Supplementary material for: Sarcopenia Is Associated With Increased Risk of Abnormal Sleep Duration in the Older People: A 10‐Year Cohort Study From China
Source: J Cachexia Sarcopenia Muscle. 2025 Jul 26;16(4):e70017. doi: 10.1002/jcsm.70017 (PMC12321970; doi:10.1002/jcsm.70017)
Supplement: Supplementary file 1 — Data S1. Supplementary Information [file JCSM-16-e70017-s001.docx]

[s1] Wang DXM, Yao J, Zirek Y, Reijnierse EM, Maier AB. Muscle mass, strength, and physical performance predicting activities of daily living: a meta-analysis. J Cachexia Sarcopenia Muscle. 2020 Feb;11(1):3-25.

[s2] Papadopoulou SK. Sarcopenia: A Contemporary Health Problem among Older Adult Populations. Nutrients. 2020;12(5):1293.

[s3] Damluji AA, Alfaraidhy M, AlHajri N, et al. Sarcopenia and Cardiovascular Diseases. Circulation. 2023;147(20):1534-1553.

[s4] Yang J, Jiang F, Yang M, Chen Z. Sarcopenia and nervous system disorders. J Neurol. 2022 Nov;269(11):5787-5797.

[s5] Locquet M, Beaudart C, Delandsheere L, Reginster JY, Bruyère O. Subjective Sleep Quality among Sarcopenic and Non-Sarcopenic Older Adults: Results from the SarcoPhAge Cohort. J Frailty Aging. 2018;7(3):176-181.

[s6] Hu K, Li W, Zhang Y, Chen H, Bai C, Yang Z, et al. Association between outdoor artificial light at night and sleep duration among older adults in China: A cross-sectional study. Environ Res. 2022 Sep;212(Pt B):113343.

[s7] Ong JC, Crawford MR, Wallace DM. Sleep Apnea and Insomnia: Emerging Evidence for Effective Clinical Management. Chest. 2021;159(5):2020-2028.
